# Addition of silver diamine fluoride to restorative materials: effect on microhardness and microleakage

**DOI:** 10.2340/biid.v12.43074

**Published:** 2025-02-14

**Authors:** Arshjot Singh Basra, Shweta Sedani, Lavannya Phaye, Rohan Khetan

**Affiliations:** Department Of Conservative Dentistry and Endodontics, Sharad Pawar Dental College and Hospital, Datta Meghe Institute of Higher Education and Research, Wardha, India

**Keywords:** Caries, composite, glass ionomer cement, silver diamine fluoride

## Abstract

**Purpose:**

The purpose of this study was to evaluate the effect of silver diamine fluoride (SDF) on microhardness and microleakage of composite and glass ionomer cement restorations.

**Materials and methods:**

Cavities were prepared in 28 extracted teeth and restored according to four regimens (GIC, composite, GIC and SDF conditioned, composite and SDF conditioned). The restored teeth were *thermocycled* and autoclaved and then exposed to cariogenic challenge (inoculated with *Lactobacillus acidophilus* and *Streptococcus mutans*) for 30 days. The restored teeth were sectioned mesio-distally. One half was subjected to microleakage testing (dye penetration using 2% methylene blue) and the other to Vickers hardness test.

**Results:**

Group 4 SDF + Composite had the highest mean microleakage at 1.59 ± 0.02 (mm), and Group 1 (GIC) and Group 3 (Composite) had the lowest mean microleakage (0.63 ± 0.009 mm and 0.63 ± 0.02 mm, respectively). The highest mean microhardness (HV) was observed in Group 3 (Composite) at 327 ± 2.16 and lowest in Group 2 (GIC + SDF) at 283 ± 1.95. Results were statistically significant with a *p* value < 0.05.

**Conclusion:**

SDF conditioning negatively impacted microhardness and increased microleakage, which could negatively impact the longevity of restorations. Therefore, in this study, SDF was not suitable as a treatment to decrease the occurrence of secondary caries.

## Introduction

Secondary caries is ‘Lesions along the margin of existing restorations’. Secondary caries is the most prevalent cause of replacing and repairing existing restorations [1]. This fact has driven the development of anti-cariogenic restorative materials like glass ionomer cement. However, studies have revealed that the antibacterial effect of the fluoride released is limited and insufficient to inhibit secondary caries development [2].

Silver diamine fluoride (SDF), a transparent liquid that combines the antibacterial benefits of silver with the remineralising effects of fluoride, is commonly advised for children who are at high risk of acquiring caries, such as those who live in poor settings or developing nations [3]. Atraumatic restorative treatment (ART), which involves the removal of demineralised tooth structure by the use of hand instruments and is mainly aimed at increasing the accessibility of treatment in isolated areas, can benefit if SDF is proven efficient in arresting caries, as it can be done in areas where electricity is scarce [4]. SDF conditioning, when combined with ART, can significantly improve treatment in isolated areas as it can be done without the need for administration of local anaesthetic, limiting the need to visit the hospital on recurrent occasions and bringing down the cost of treatment and treatment time.

In 2014, SDF was approved by the US Food and Drug Administration to manage dentinal sensitivity. Silver-modified atraumatic restorative treatment (SMART) is a recent advancement that effectively inhibits carious processes without invasive removal of additional healthy tooth structure. This indicates a possible role SMART can have in preventing secondary caries.

*Lactobacillus acidophilus* and *Streptococcus mutans* are frequently regarded as the most significant cariogenic bacteria linked with dentinal caries [5]. Several studies have shown that SDF can inhibit the growth of both of these cariogenic bacteria [5, 6]. *In vitro* studies have shown that SDF improves biofilm pH, prevents dentin demineralisation, and has antibacterial activity against cariogenic bacteria [7].

The safety of any restorative material has the utmost importance; in the case of SDF, safety does not seem to be an issue as it was used first in Japan around the 1970s, almost 80 years ago [8], which has been one of the most technologically advanced countries in the world. However, the incidence of staining of teeth has been reported with the use of SDF [9] and is thus among the challenges faced with using SDF.

Llodra et al. conducted a study on 425 children divided into two groups. The study aimed to observe the effect of SDF on deciduous teeth of children aged 6 years in arresting caries. The first group of the study received SDF at 38% concentration at an interval of 6 months for a total of 36 months, while a second group served as control. At the end of the study, the mean value in the two groups of new decayed surfaces appearing in primary teeth during the study was 0.29 in the SDF group and 1.43 in the control group. This study indicated the possible anti-cariogenic effect of SDF on human dentition [10].

Mei et al. conducted a study in which *S. mutans* and *L. acidophilus* were used to immerse 30 human dentine blocks under anaerobic conditions for 7 days to produce carious lesions. They were equally split into a test and control group; SDF and water were applied topically to the respective groups. The biofilm formed was later evaluated on different parameters, one being colony forming unit (CFU) of *L. acidophilus* and *S. mutans*. The CFU was observably less in the group in which SDF conditioning was done as compared to the control group [5].

Chu et al. conducted a study that included 375 children with carious teeth in the maxillary jaw, excluding only carious in posterior teeth aged between 3 and 5 years of age. Subjects were divided into five groups, SDF conditioning made annually in the 1st and 2nd groups, the 3rd and 4th groups being exposed to sodium fluoride varnish every 3 months, and 5th group acting as a control. The study was carried out for 2 years and 6 months. The five groups’ relative mean number of arrested carious tooth surfaces were 2.5, 2.8, 1.5, 1.5, and 1.3 (*p* value less than 0.001) [8]. These results indicated a positive correlation between SDF conditioning and the arrest of caries.

Studies have shown clinical success in arresting dentinal caries with the use of SDF [5, 7, 8, 10]. The effect of SDF on individual factors that affect the success of restorations, such as microleakage and microhardness, is still shrouded in much mystery. This study aimed to evaluate the effect of SDF on microleakage and microhardness of glass ionomer cement and composite resin restorations. The null hypotheses tested were that SDF conditioning would decrease the microleakage, while microhardness would not be affected.

## Materials and methods

This study was conducted at Sharad Pawar Dental College in Wardha, Maharashtra, India, in the Department of Paediatric and Preventive Dentistry between January 2023 and November 2023. Ethical approval to conduct the study was received from the Institutional Ethics Committee (IEC ref number. DMIHER(DU)/IEC/2022/1179)*.*

### Sample size

The sample size was calculated by comparing outer lesion depths between two groups as per reference article [11].

Group 1 (SDF + GIC) with a mean of 156 and standard deviation (SD) of 45.

Group 2 (GIC) with a mean of 235 and SD of 33.

The calculation is based on the following formula for comparing two means:

*n* = ((*Z*α + *Z*β)² * (2 * SD²)) / Δ²

Where:

*n* = required sample size per group.

*Z*α = *Z* value for the chosen significance level (α), for a two-tailed test (1.96 for α = 0.05).

*Z*β = *Z* value for the desired power (1.645 for 95% power).

SD = pooled SD of the two groups.

Δ = difference in means between the two groups

The calculated sample size for this study, based on the mean difference of 79 and a pooled SD of 39.46, with 95% power, is seven samples per group.

### Material selection and cavity preparation

A total of 28 intact extracted teeth were collected. Inclusion criteria: healthy, intact permanent premolars extracted for orthodontic treatment and patients between the age of 10 and 35 years. Exclusion criteria: diabetic patients, cancer patients, and osteoporosis patients. The teeth were cleaned thoroughly of any residual debris or blood.

A Type 2 cavity of approximately the same size was prepared on each tooth using an air rotor (Waldent Premium Plus, China) with diamond bur (SuperEndo Cavity Preparation Burs Kit, China) under water cooling. All cavities were cleaned with 10% polyacrylic acid (GC Gold Label 2, Japan) and divided into the following four groups with seven samples in each group:

Group 1: Cavity restored with glass ionomer cement (GC Gold Label 2, Japan).

Group 2: Cavity filling with SDF (Fagamin SDF 38%, Tedequim, Argentina) for 3 minutes, followed by conditioning of glass ionomer cement.

Group 3: Cavity surface treated with a seventh-generation bonding agent (Prime Dental Restorite Bond 7th Generation, India), followed by filling with composite resin (Restorite Micro Hybrid composite B1, Prime Dental Products, India).

Group 4: Cavity conditioned with SDF for 3 minutes, followed by treatment with a seventh-generation bonding agent and filling with composite resin.

### Thermocycling and autoclaving

All restored teeth were coated with nail varnish (acid resistant), excluding a zone of about 1 mm around the restoration. To mimic the condition of aged restorations, the restored teeth were thermocycled for 400 cycles in decontaminated water. Immersion was done at 50–60°C and 5–15°C, with a 30-second dwelling time for each immersion and a 15-second period between the immersions. The teeth were sterilised by the use of an autoclave before exposure to challenge with cariogenic bacteria.

### Cariogenic challenge

*L. acidophilus* and *S. mutans* isolated colonies grown on blood agar plates were transferred to tubes containing Robertson’s Cooked Meat broth and 5.0% sucrose and inoculated for 24 hours, anaerobically at a temperature of 37.0°C. Each tooth was drenched in a test tube containing 10.00 mL of prepared bacterial medium and maintained in this medium at 37.0°C for 30 days anaerobically; the prepared medium was changed every 48 hours. During the inoculation period, Gram staining of the medium was carried out to inspect for contaminants.

### Assessment and data collection

#### Microleakage test

Each treated tooth, was sectioned mesio-distally (Buehler’s Petro Thin Sectioning system, USA). The procedure included dye penetration using 2% methylene blue (Dr.Willmar Schwabe India Pvt. Ltd) in the sectioned samples for 6 minutes, followed by cleaning with distilled water. Prepared sections were observed under a stereo microscope (Magnus MLX-B Plus, Germany) at 10 × magnification, and digital images were obtained using C- mount microscope camera (Magnus MAGCAM DC-5 CMOS, Germany) at 40 × magnification (6000 μm field of view) and pixel size of 2.2 × 2.2 (um). Digital images were analysed based on image properties (6000 μm field of view and pixel size of 2.2 × 2.2 um) by image analysis system (ImageJ analysis System, v. 1.50, National Institute of Health, USA) to calculate microleakage by measuring the depth of dye penetration in millimeter (mm) along restoration margins.

#### Microhardness test

Each treated tooth, was sectioned mesio-distally (Buehler’s Petro Thin Sectioning system, USA). The procedure included embedding the sectioned samples in acrylic resin (ClaroFast, Struers, Denmark) and performing the Vickers test at a load of 100 g (indenting the prepared samples with a diamond Pyramid of specified load for 10–15 seconds), taking reference standard as ISO 6507. Final values were recorded in Vickers hardness number (HV) by calculating the indentation area.

### Statistical analysis

The statistical analysis was conducted using IBM Statistical Package for the Social Sciences (SPSS) Statistics for Windows, Version 23 (Released 2015; IBM Corp., Armonk, New York, United States) with a significance threshold of < 0.05. One-way analysis of variance (ANOVA) with the Tukey test was performed to compare the effects of restorative materials and SDF (as two predicting variables) on microleakage and microhardness.

## Results

### Microleakage

**Figure 1:**
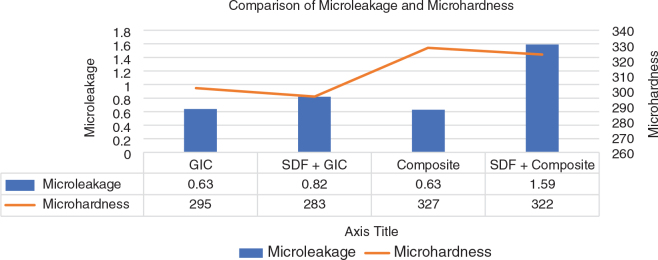
Comparison of mean microleakage and mean microhardness in the four groups. SDF: silver diamine fluoride.

With regard to microleakage, ANOVA found differences between the groups to be statistically significant (*p* < 0.001), and the Tukey test found that results of the intergroup comparison were statistically significant (*p* < 0.001) except when comparing Groups 1 and 3 (GIC vs composite). The lowest penetration of dye was observed in Group 3 (composite), followed by Group 1 (GIC) and Group 2 (SDF + GIC), respectively, and the highest penetration was observed in Group 4 (SDF + composite). On dividing the groups between SDF-conditioned and non-SDF-conditioned groups based on data, a high increase in the degree of dye penetration was seen in the SDF-conditioned group as compared to that of the non-SDF conditioned (Tables 1, 2 and Figure 1).

**Table 1:** Comparison of mean microleakage and mean microhardness among the four groups.

Groups	Microleakage (mm)	Microhardness (HV)
GIC	0.63 ± 0.009	295 ± 6.68
SDF + GIC	0.82 ± 0.02	283 ± 1.95
COMPOSITE	0.63 ± 0.02	327 ± 2.16
SDF + COMPOSITE	1.59 ± 0.02	322 ± 2.64
*P* value	< 0.001*	< 0.001*

SDF: silver diamine fluoride.

* Statistically significant.

**Table 2:** Comparison of mean microleakage between various groups.

Groups	Microleakage (mm)	*P* value
GIC VS SDF GIC	0.180 ± 0.02	0.001*
GIC VS COMPOSITE	0.005 ± 0.02	0.89
GIC VS SDF COMPOSITE	0.95 ± 0.03	0.001*
SDF GIC VS COMPOSITE	0.19 ± 0.01	0.001*
SDF GIC VS SDF COMPOSITE	0.77 ± 0.03	0.001*
COMPOSITE VS SDF COMPOSITE	0.96 ± 0.04	0.001*

SDF: silver diamine fluoride.

* Statistically significant.

### Microhardness

With regard to microhardness, ANOVA found differences between the groups to be statistically significant (*p* < 0.001), and the Tukey test found that the results of the intergroup comparison were statistically significant (*p* < 0.001) except when comparing Groups 3 and 4 (composite vs SDF + composite). the lowest microhardness was seen in Group 2 (SDF + GIC), followed by Group 1 (GIC) and Group 4 (SDF + composite), respectively and with the highest value being recorded in Group 3 (composite). On dividing the groups based on the material used, a clear increase in microhardness was observed in the group where GIC was used compared to composite (Tables 1, 3 and Figure 1).

**Table 3:** Comparison of mean microhardness among the four groups.

Groups	Microhardness (mm)	*P* value
COMPOSITE VS SDF COMPOSITE	5.0 ± 0.57	0.19
GIC VS COMPOSITE	32 ± 5.03	0.001*
COMPOSITE VS SDF GIC	44 ± 3.21	0.001*
SDF COMPOSITE VS GIC	27 ± 4.58	0.001*
SDF GIC VS SDF COMPOSITE	39 ± 2.94	0.001*
GIC VS SDF GIC	12 ± 5.19	0.001*

SDF: silver diamine fluoride.

* Statistically significant.

## Discussion

In this study, the effect of SDF conditioning in conjunction with different restorative materials (GIC and composite) on microleakage and microhardness was evaluated. It was observed that the SDF conditioning negatively affected both parameters. Secondary caries, a highly prevalent cause for restoring existing restorations, has mandated the research of better anti-cariogenic restorative materials [12]. This has led to the development of materials such as glass ionomer cement and composites, which have improved the restoration prognosis in clinical practice. However, GIC and composite restorations lack the required antibacterial action to inhibit secondary caries development. A previous study has shown that remineralisation assisted by the fluoride release or that occurring naturally is insufficient when the bacterial load is high and hence unable to arrest the caries development [12, 13]. SDF has shown the ability to remineralise the enamel as well as dentin by the action of its fluoride component and antibacterial effect on biofilm, even though the effectiveness of SDF is still a matter of research [6, 14, 15].

Several methods have been used to study the microleakage of various fluids and bacteria along the prepared cavity walls and restorative material. In the study conducted, we observed that microleakage or depth of dye penetration was highest in Group 4 (SDF + composite). These findings indicate that SDF conditioning could hamper the bond strength of the bonding agent. This is in accordance with the studies of Lutgen et al., Soeno et al., Kucukyilmaz et al., and Koizumi et al. [13, 16, 17, 18]. However, interference with the bonding agent cannot explain the high value of microleakage in GIC, as bonding agents have no role in GIC restorations. This indicates that SDF conditioning on dentine may result in inferior bond strength when followed by GIC restoration. These results are in accordance with Knight et al. [19]. These findings are consistent with prior studies on the effect of SDF on the bond strength of restorative materials and can be attributed to the fact that the pH of SDF is higher than the etching/conditioning agents used, which hampers their effect and prevent intimate adaptation of dentine and restorative materials. Thus, the finding that SDF conditioning may hamper bond strength of GIC, as well as of composite, could be a major setback in the use of SDF, as microleakage is an important factor in the development of secondary caries and the success of restorative treatment in the long term.

Composite had the highest mean microhardness and GIC the lowest (Figure 1). This superior microhardness of composite is in accordance with Boyer et al. and Kwon et al. [20, 21] and can be explained by the higher inorganic filler content volume of the composite. Whereas SDF conditioning resulted in a significant drop in microhardness of the GIC, a slight, but non-significant decrease in hardness was also seen for the composite. These findings are in accordance with Delbem et al. [22] and support their finding that SDF conditioning adversely affected fluoride retained in different layers of enamel, which made enamel prone to cariogenic action. Hence, these microhardness results signify a clear advantage of composite over GIC as a restorative material.

The results of this study necessitate the rejection of the null hypothesis since SDF conditioning was associated with increased microleakage and reduced microhardness. Relation between factors like microhardness, antibacterial properties, polymerisation shrinkage, microleakage, and numerous others need to be assessed, and further research is required on different materials, including SDF, glass ionomer cement, and composite, to obtain sufficient data to conclude on ideal restorative material to reduce secondary caries prevalence. However, in this study, we got a step closer to such an aim as the results showed that SDF conditioning negatively impacted microhardness and microleakage, two factors that could negatively impact the longevity of the restoration. Therefore, in this study, SDF was not suitable for decreasing the occurrence of secondary caries.

## Conclusions

The development of secondary caries is a multifactorial process. This study found that SDF conditioning negatively impacted microhardness and microleakage, possibly due to high pH and low fluoride retention of enamel associated with the use of SDF. This could negatively impact the longevity of restorations. Thus, SDF conditioning would be a poor choice for patients at high risk of caries. However, the effect of SDF in inhibiting the bacterial biofilm is still a matter of research.

## Data Availability

Not applicable.

## References

[1] Gordan VV, Riley JL, Geraldeli S, Rindal DB, Qvist V, Fellows JL, et al. Repair or replacement of defective restorations by dentists in the dental practice-based research network. J Am Dent Assoc. 2012;143:593–601. 10.14219/jada.archive.2012.0238 22653939 PMC3368503

[2] Mjör IA, Toffenetti F. Secondary caries: a literature review with case reports. Quintessence Int [Internet]. 2000;31(3):165–79. Available from: https://pubmed.ncbi.nlm.nih.gov/11203922/ [cited 21/09/2024] 11203922

[3] Sharma G. Approaches to arresting dental caries: an update. J Clin Diagn Res. 2015;9(5):ZE08–11. 10.7860/JCDR/2015/12774.5943 26155592 PMC4484184

[4] Giacaman RA, Muñoz-Sandoval C, Neuhaus KW, Fontana M, Chałas R. Evidence-based strategies for the minimally invasive treatment of carious lesions: review of the literature. Adv Clin Exp Med. 2018;27:1009–16. 10.17219/acem/77022 29962116

[5] Mei M-L, Chu C-H, Low K-H, Che C-M, Lo E-C-M. Caries arresting effect of silver diamine fluoride on dentine carious lesion with S. mutans and L. acidophilus dual-species cariogenic biofilm. Med Oral Patol Oral Cir Bucal. 2013;18(6):e824–31. 10.4317/medoral.18831 23722131 PMC3854072

[6] Mei ML, Li Q, Chu C-H, Lo E-M, Samaranayake LP. Antibacterial effects of silver diamine fluoride on multi-species cariogenic biofilm on caries. Ann Clin Microbiol Antimicrob. 2013;12:4. 10.1186/1476-0711-12-4 23442825 PMC3599989

[7] Chu CH, Mei L, Seneviratne CJ, Lo ECM. Effects of silver diamine fluoride on dentine carious lesions induced by *Streptococcus mutans* and *Actinomyces naeslundii* biofilms. Int J Paed Dentistry. 2012;22:2–10. 10.1111/j.1365-263X.2011.01149.x 21702854

[8] Chu CH, Lo ECM, Lin HC. Effectiveness of silver diamine fluoride and sodium fluoride varnish in arresting dentin caries in Chinese pre-school children. J Dent Res. 2002;81:767–70. 10.1177/0810767 12407092

[9] Garg S, Sadr A, Chan D. Potassium iodide reversal of silver diamine fluoride staining: a case report. Oper Dent. 2019;44:221–6. 10.2341/17-266-S 31046649

[10] Llodra JC, Rodriguez A, Ferrer B, Menardia V, Ramos T, Morato M. Efficacy of silver diamine fluoride for caries reduction in primary teeth and first permanent molars of schoolchildren: 36-month clinical trial. J Dent Res. 2005;84:721–4. 10.1177/154405910508400807 16040729

[11] Mei ML, Zhao IS, Ito L, Lo EC-M, Chu C-H. Prevention of secondary caries by silver diamine fluoride. Int Dent J. 2016;66:71–7. 10.1111/idj.12207 26689611 PMC9376532

[12] Featherstone JDB. Remineralization, the natural caries repair process – the need for new approaches. Adv Dent Res. 2009;21:4–7. 10.1177/0895937409335590 19717404

[13] Mei ML, Ito L, Cao Y, Li QL, Lo ECM, Chu CH. Inhibitory effect of silver diamine fluoride on dentine demineralisation and collagen degradation. J Dent. 2013;41:809–17. 10.1016/j.jdent.2013.06.009 23810851

[14] Chu CH, Lo ECM. Microhardness of dentine in primary teeth after topical fluoride applications. J Dent. 2008;36:387–91. 10.1016/j.jdent.2008.02.013 18378377

[15] Lutgen P, Chan D, Sadr A. Effects of silver diammine fluoride on bond strength of adhesives to sound dentin. Dent Mater J. 2018;37:1003–9. 10.4012/dmj.2017-401 30224603

[16] Soeno K, Taira Y, Matsumura H, Atsuta M. Effect of desensitizers on bond strength of adhesive luting agents to dentin. J Oral Rehabil. 2001;28:1122–8. 10.1046/j.1365-2842.2001.00756.x 11874511

[17] Kucukyilmaz E, Savas S, Akcay M, Bolukbasi B. Effect of silver diamine fluoride and ammonium hexafluorosilicate applications with and without Er:YAG laser irradiation on the microtensile bond strength in sound and caries‐affected dentin. Lasers Surg Med. 2016;48:62–9. 10.1002/lsm.22439 26729655

[18] Koizumi H, Hamama HH, Burrow MF. Effect of a silver diamine fluoride and potassium iodide-based desensitizing and cavity cleaning agent on bond strength to dentine. Int J Adhes Adhes. 2016;68:54–61. 10.1016/j.ijadhadh.2016.02.008

[19] Knight G, McIntyre J, Mulyani. The effect of silver fluoride and potassium iodide on the bond strength of auto cure glass ionomer cement to dentine. Aust Dent J. 2006;51:42–5. 10.1111/j.1834-7819.2006.tb00399.x 16669476

[20] Boyer DB, Chalkley Y, Chan KC. Correlation between strength of bonding to enamel and mechanical properties of dental composites. J Biomed Mater Res. 1982;16:775–83. 10.1002/jbm.820160604 7174707

[21] Kwon YH, Kwon T-Y, Ong JL, Kim K-H. Light-polymerized compomers: coefficient of thermal expansion and microhardness. J Prosthet Dent. 2002;88:396–401. 10.1067/mpr.2002.128121 12447216

[22] Delbem ACB, Bergamaschi M, Sassaki KT, Cunha RF. Effect of fluoridated varnish and silver diamine fluoride solution on enamel demineralization: pH-cycling study. J Appl Oral Sci. 2006;14:88–92. 10.1590/S1678-77572006000200005 19089037 PMC4327448

